# The Relationship of ST Segment Changes in Lead aVR with Outcomes after Myocardial Infarction; a Cross Sectional Study

**Published:** 2017-06-30

**Authors:** Mohammad Reza Beyranvand, Mohammad Assadpour Piranfar, Mohammadreza Mobini, Mehdi Pishgahi

**Affiliations:** 1 Department of Cardiology, Taleghani Hospital, Shahid Beheshti University of Medical Sciences, Tehran, Iran.; 2 Department of Cardiology, Shohadaye Tajrish Hospital, Shahid Beheshti University of Medical Sciences, Tehran, Iran.

**Keywords:** Myocardial infarction, electrocardiography, patient outcome assessment, st elevation myocardial infarction

## Abstract

**Introduction:**

Among the 12 leads studied in electrocardiography (ECG), lead aVR can be considered as the most forgotten part of it since no attention is paid to it as the mirror image of other leads. Therefore, the present study has been designed with the aim of evaluating the prevalence of ST segment changes in lead aVR and its relationship with the outcome of these patients.

**Methods:**

In this retrospective cross sectional study medical profiles of patients who had presented to emergency department with the final diagnosis of myocardial infarction (MI) in a 4-year period were evaluated regarding changes of ST segment in lead aVR and its relationship with in-hospital mortality, the number of vessels involved, infarct location and cardiac ejection fraction.

**Results:**

288 patients with the mean age of 59.00 ± 13.14 (18 – 91) were evaluated (79.2% male). 168 (58.3%) patients had the mentioned changes (79.2% male). There was no significant relationship between presence of ST changes in lead aVR with infarct location (p = 0.976), number of vessels involved (p = 0.269) and ejection fraction on admission (p = 0.801). However, ST elevation ≥ 1 mv in lead aVR had a significant relationship with mortality (Odds = 7.72, 95% CI: 3.07 – 19.42, p < 0.001). Sensitivity, specificity, positive and negative predictive values and positive and negative likelihood ratios of ST elevation ≥ 1 for prediction of in-hospital mortality were 41.66 (95% CI: 22.79 – 63.05), 91.53 (95% CI: 87.29 – 94.50), 31.25 (95% CI: 16.74 – 50.13), 94.44 (95% CI: 90.65 – 96.81), 0.45 (95% CI: 0.25 – 0.79), and 0.05 (95% CI: 0.03 – 0.09), respectively.

**Conclusion:**

Based on the results of the present study, the prevalence of ST segment changes in lead aVR was estimated to be 58.3%. There was no significant relationship between these changes and the number of vessels involved in angiography, infarct location and cardiac ejection fraction. However, presence of ST elevation ≥ 1 in lead aVR was associated with 8 times increase in in-hospital mortality risk.

## Introduction

Chest pain caused by myocardial infarction (MI) is among the most common causes of patients visiting emergency departments (EDs). There has been a prediction of 80% increase in cases affected with problems related to coronary arteries in developing countries such as India, Latin America Region, Middle East, and African Sahara by 2020 (1, 2). Predicting the outcome of these patients can be helpful in more proper triage, giving information to their relatives, and more accurate care for the more severe cases. The final outcome of these patients depends on various factors such as age, size and place of the infarction, residual left ventricular function, presence of underlying illnesses, and delay in reperfusion of affected arteries (3-6).

Numerous studies have been done regarding the relationship between electrocardiogram (ECG) evidence with angiography findings and final outcome of these patients and among them, ST segment changes in lead aVR has been associated with poor outcome of patients with MI during their hospitalization period (7-9). 

In a study by Kukla et al. patients who had ST-elevation in lead aVR, faced in hospital death 1.5 times those with ST-depression and about 30 times those without changes in ST segment (10).

In addition, findings of Senaratne et al. revealed that mortality rate was 16 times higher in patients who had MI with ST depression in lead aVR (11). They expressed that although ST segment depression in lead aVR is difficult to diagnose, it can be good evidence of ischemia or injury to apex and infrolateral regions of the heart.

Presence of ST segment depression is indicative of a larger area on the cardiac muscle being involved and somehow shows the need for more invasive interventions (12, 13). Additionally, ST elevation in lead aVR has been associated with increased probability of recurrent infarction, developing cardiac failure and increased need for bypass surgery of coronary arteries (12). A study by Kosuge et al., indicated 78% sensitivity and 86% specificity of more than 0.05 mV ST segment elevation in aVR lead for predicting involvement of all 3 cardiac vessels (14). Until now, no similar studies have been carried out on Iranian patients in this field; therefore, the present study has been designed with the aim of evaluating the relationship of ST segment elevation in lead aVR with the final outcome of the patients with MI presenting to ED.

## Methods

***Study design***

In this retrospective cross sectional study medical profiles of patients who were hospitalized in Taleghani Hospital, Tehran, Iran, during the time between 2012 and 2015 with the final diagnosis of MI were evaluated with the aim of assessing the prevalence of ST segment changes in lead aVR and its relationship with the outcome of studied patients. The study was approved by the ethics committee of Shahid Beheshti University of Medical Sciences. Researchers adhered to the principles of Helsinki declaration and keeping patients’ data confidential.

***Participants***

Patients who had been hospitalized with the final diagnosis of MI in the mentioned period were evaluated without any age or sex limitations. Inclusion criteria consisted of time interval of less than 24 hours between chest pain and presenting to ED, confirmation of MI based on clinical findings and raise in cardiac enzymes (CPKMB or troponin) in the initial 48 hours of admission. Patients with a history of coronary artery bypass grafting (CABG), history of heart blocks including left bundle branch block (LBBB) and right bundle branch block (RBBB), and those with a pace maker as well as cases where the information was not completely available were excluded from the study.

***Data gathering***

Data gathering was done using a pre-designed checklist by referring to patients’ medical profile. A senior cardiology resident was in charge of gathering patients’ data. Studied variables included demographic data (age and sex), vital signs of the patient on admission to ED, medical history, drug history, left ventricle ejection fraction based on echocardiography findings, ECG findings regarding changes of ST segment in lead aVR, angiography findings regarding number and location of coronary artery involvement and finally, mortality of the patients. Locating the infarct zone was done based on ECG findings and Echocardiography confirmation.

Statistical analysis

The sample size required for performing the study was estimated as 288 cases by considering the probability of changes being present in lead aVR to be 25%, type 1 error of 5%, 80% power, and d = 10%. SPSS21 software was used for statistical analysis. To report variables, descriptive statistics such as mean ± standard deviation or frequency and percentage were used in tables and a chart. To compare qualitative variables chi square or Fisher’s exact tests and for quantitative cases, t-test was applied. Additionally, sensitivity, specificity, positive and negative predictive values and positive and negative likelihood ratio as well as area under the receiver operating characteristic (ROC) curve of ST elevation ≥ 1 in lead aVR were calculated and reported with 95% confidence interval using Med Calc software. P < 0.05 was considered as significance level.

## Results

***Baseline characteristics***

**Figure 1:**
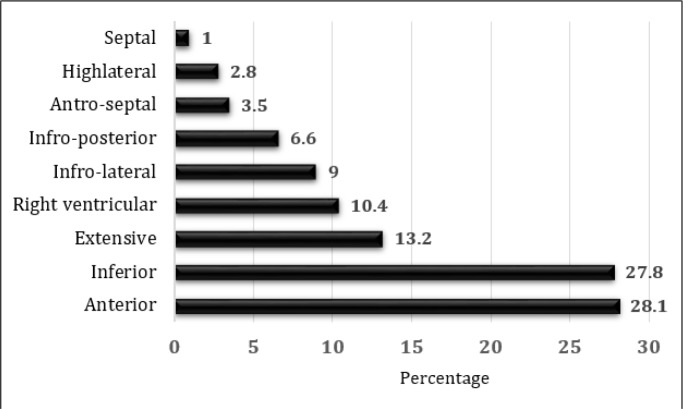
Infarct Location based on electrocardiographic findings.

288 patients with the mean age of 59.00 ± 13.14 (18 – 91) were evaluated (79.2% male). Mean duration of hospitalization was 6.11 ± 4.82 (1 – 45) days. 247 (85.8%) of the MI cases were ST elevation MI (STMI) and 41 (14.2%) cases had non STMI. Table and figure 1 show the baseline characteristics of the studied patients. More than half of the patients were over 60 years old (50.3%). Only, 11 (3.8%) patients had unstable hemodynamics on presentation to ED and all but 10 (3.5%) patients had degrees of decrease in cardiac ejection fraction on admission to ED. Based on the results of angiography, 93 (32.3%) cases had three vessel involvement, 85 (29.5%) had two vessel, 63 (21.9%) had single vessel involvement and in 4 cases angiography was normal. Table 2 depicts the location of vascular lesion based on angiography results. 168 (58.3%) patients had changes of ST segment in lead aVR (79.2% male). Findings regarding changes in ST segment elevation in lead aVR are summarized in table 3. There was no relationship between presence of these changes with age (p = 0.260), sex (p = 0.977) and type of MI (p = 0.247).

***Relationship of ST segment changes with outcome***

There was no significant relationship between presence of ST changes in lead aVR with infarct location (p = 0.976), number of vessels involved (p = 0.269) and cardiac ejection fraction on admission (p = 0.801). Out of the 32 (11.1%) patients who finally died, 25 (78.1%) had ST changes in lead aVR (Odds = 2.72, 95% CI: 1.13 – 6.52, p < 0.020).

***Relationship of ST elevation ≥ 1 mv with outcome***

**Figure 2:**
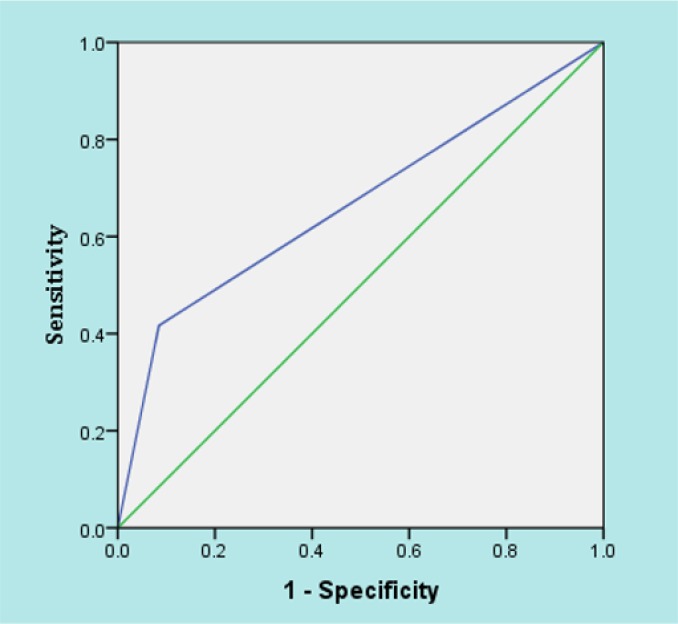
The area under the ROC curve of ST segment elevation ≥ 1 millivolt in predicting the mortality of patients with myocardial infarction.

There was no relationship between presence of ST elevation ≥ 1 mv in lead aVR with infarct location (p = 0.466), number of vessels involved (p = 0.206) and cardiac ejection fraction on admission (p = 0.369). However, it had a significant relationship with mortality (Odds = 7.72, 95% CI: 3.07 – 19.42, p < 0.001). Sensitivity, specificity, positive and negative predictive values and positive and negative likelihood ratios of ST elevation ≥ 1 mv in lead aVR for prediction of in-hospital mortality were 41.66 (95% CI: 22.79 – 63.05), 91.53 (95% CI: 87.29 – 94.50), 31.25 (95% CI: 16.74 – 50.13), 94.44 (95% CI: 90.65 – 96.81), 0.45 (95% CI: 0.25 – 0.79), and 0.05 (95% CI: 0.03 – 0.09), respectively. In addition, based on area under the ROC curve its accuracy was 0.66 (95% CI: 0.53 – 0.76) (figure 2).

***Relationship of ST depression ≥ 1 mv with outcome***

There was no significant relationship between ST depression ≥ 1 in lead aVR with infarct location (p = 0.160), number of vessels involved (p = 0.521), cardiac ejection fraction on admission (p = 0.309) and mortality (p = 0.546).

## Discussion

Based on the results of the present study, the prevalence of ST segment changes in lead aVR was estimated to be 58.3%. There was no significant relationship between these changes and the number of vessels involved in angiography, infarct location and cardiac ejection fraction. However, presence of ST elevation ≥ 1 in lead aVR was associated with 8 times increase in in-hospital mortality risk. 

ECG, as a cheap and non-invasive method, has been used for more than 70 years around the world to diagnose cardiac tissue ischemia and MI. Among the 12 leads studied in ECG, lead aVR can be considered as the most forgotten part of it since no attention is paid to it as the mirror image of other leads. In the last few decades, this lead is regaining its place as an important part of ECG among cardiologists. ST segment changes can be considered as the most important ECG finding in diagnosis and evaluation of MIs. Lead aVR is a good reference regarding things that occur in the upper and right side of the heart (15). The findings of this lead are usually covered by the information from the left leads of the heart such as aVL, II, V5, and V6; that is why it has been forgotten. Researchers Yamaji et al. in 2001 had believed that ST segment elevation changes are good indicators of ischemia in the basal parts of the interventricular septum of the heart (16). On the other hand, Gorgels et al. in Netherlands could link the elevation of ST segment in aVR with obstruction of the proximal region of the left anterior descending (LAD) artery and involvement of the first septal branch of this artery (15). Involvement of the distal parts of LAD is usually accompanied by ST segment depression in aVR.

Wong et al. in 2012 went further and suggested a U shape relationship between 30-day mortality of those with anterior MI and ST level in aVR (17). So that more than 0.5 mv ST segment depression in aVR was associated with higher mortality rate during the 30 days after occurrence of anterior MI. The last chest lead (V6) is placed on the axilla midline and a lead V7 in the posterior axillary line can show wide ischemia of the cardiac tip more clearly. This finding is indicative of the importance of the mirror image in aVR, which is more reflective of ischemia in the apex of the heart and shows the mirror image of V7 more than any other lead. This means that deeper depression in aVR in ST is a sign of higher ST elevation not only in V5 and V6 leads but also in V7 (17). Using ST segment, T wave and Q wave in lead aVR to evaluate the current or past situation of the patients such as previous or current MI has been suggested in various studies (18).

Although Wong believes that ST segment changes in lead aVR are not significantly related to mortality of MI patients, in 2012 Kukla et al. announced that among 320 patients with inferior MI who had ST segment changes in various leads of ECG, these changes in lead aVR had occurred in half the patients and had a significant relationship with poor prognosis for their problem (10, 17).

**Table 1:** Baseline characteristics of the studied patients

**Variable **	**N (%)**
**Sex**	
Male	228 (79.2)
Female	60 (20.8)
**Age (year)**	
15 - 30	6 (2.1)
30 - 45	26 (9.0)
45 - 60	111 (38.5)
≥ 60	145 (50.3)
**Hemodynamic **	
Stable	275 (95.5)
unstable	11 (3.8)
**Medical history**	
Smoking	129 (44.8)
Diabetes mellitus	78 (27.1)
Hypertension	119 (41.3)
Hyperlipidemia	85 (29.5)
Prior MI	23 (0.8)
Prior CVA	21 (7.3)
**Drug History**	
Calcium channel blockers	10 (3.5)
β blockers	252 (78.5)
Nitrates	158 (54.9)
Aspirin	253 (87.8)
Clopidogrel	230 (79.9)
Statins	255 (88.5)
ACEI/ARB	240 (83.3)
**LVEF (%)**	
≥ 55	10 (3.5)
45 - 55	138 (47.9)
35 – 45	78 (25.7)
< 35	38 (13.2)

MI: myocardial infarction; CVA: cardiovascular accident; LVEF: left ventricular ejection fraction; ACEI/ARB: angiotensin-converting enzyme inhibitor /angiotensin-receptor blocker.

**Table 2:** Location of vascular lesion based on angiographic findings

**Arteries**	**N (%)**
**Left main**	1 (0.3)
**Left anterior descending**	
Proximal	41 (14.2)
Medial	80 (27.8)
Distal	5 (1.7)
**Left circumflex**	
Proximal	4 (1.4)
Medial	11 (3.8)
Distal	2 (0.7)
**Right coronary**	
Proximal	30 (10.4)
Medial	33 (11.5)
Distal	10 (3.5)
**Others**	25 (8.7)

**Table 3:** ST segment changes in lead aVR of the studied patients

**ST changes**	**N (%)**
**Normal**	
Isoelectric	116 (40.3)
**Elevation**	
0.5 - 1	79 (27.4)
≥ 1	24 (8.3)
**Depression**	
0.5 - 1	53 (18.4)
≥ 1	12 (4.1)

In the present study, a significant statistical relationship was found between ST segment changes in lead aVR and mortality of the patients. The higher the elevation, the higher the prevalence of mortality. ST segment depression and isoelectric level of the segment, respectively, were associated with lower levels of mortality. Some studies have expressed the higher importance of ST segment elevation in aVR compared with this segment’s depression (12). Meanwhile, Kukla et al. in their study on 320 individuals observed that patients with inferior wall MI, in case of ST elevation in aVR had a mortality rate 1.5 times those who had ST depression and 30 times those with isoelectric ST in aVR (10). While, Senaratne in 2003 showed that ST segment depression in aVR was associated with 16 times more patient mortality compared to other cases (11).

Regarding the anatomy of coronary arteries’ involvement and its relationship with changes in lead aVR, no significant relationship was found in our study; however, ST segment elevation in aVR was accompanied with higher involvement of all 3 coronary arteries. On the other hand, although not statistically significant, proximal obstruction of LAD and medial obstruction of the left circumflex artery was accompanied by higher mortality in patients. 

Mortality rate in the present study was 11.1%, which is higher than the mean mortality rate reported during hospital stay in a meta-analysis done by Sorita et al in 2014 (7%) (19). This meta-analysis was carried out on 48 research studies related to the topic and more than 1.5 million patients, and reported mortality of MI patients to be 12% during the 30 days after occurrence of MI.

Although this study was done on 288 MI patients and the sample size and power of the study were acceptable, its only statistically significant finding was the relationship between ST segment elevation in lead aVR and in-hospital mortality, which is of course highly important, and ST changes in lead aVR did not significantly change with LVEF changes and coronary artery involvement and its location. 

It seems that performing prospective cohort studies by considering all of the identified risk factors in prediction of MI patients’ outcome can give a more accurate picture of the role of lead aVR findings in prediction of outcome and estimation of location or extent of the necrosis occurring following ischemia.

***Limitation:***

This study was done retrospectively by evaluating patients’ medical profiles and therefore, had the natural limitations associated with these studies such as missing information and not being sure of the accuracy of the reports. Other known risk factors of patient outcome were not evaluated in this study, thus, multivariate analysis for identifying independent factors could not be performed.

## Conclusion

Based on the results of the present study, the prevalence of ST segment changes in lead aVR was estimated to be 58.3%. There was no significant relationship between these changes and the number of vessels involved in angiography, infarct location and cardiac ejection fraction. However, presence of ST elevation ≥ 1 mv in lead aVR was associated with 8 times increase in in-hospital mortality risk. 
